# Post-tuberculosis and postinfected bronchiectasis: data from global registries

**DOI:** 10.36416/1806-3756/e20250153

**Published:** 2025-07-22

**Authors:** Grace Oscullo, Miguel-Angel Martinez-García, Rosella Centis, Lia D’Ambrosio, Jose Daniel Gómez-Olivas, Giovanni Battista Migliori

**Affiliations:** 1. Servicio de Neumología e Instituto de Investigación La Fe - IISLAFE - Hospital Universitario y Politécnico La Fe, Valencia, España.; 2. Centro de Investigación Biomédica en Red de Enfermedades Respiratorias, Instituto de Salud Carlos III, Madrid, España.; 3. Servizio di Epidemiologia Clinica delle Malattie Respiratorie, Istituti Clinici Scientifici Maugeri - IRCCS - Tradate, Italia.; 4. Public Health Consulting Group, Lugano, Switzerland.

Evidence on the relationship between bronchiectasis and post-tuberculosis lung disease (PTLD) is scant, and the global prevalence of post-tuberculosis bronchiectasis has yet to be investigated.

Bronchiectasis is characterized by an increased bronchial lumen diameter relative to that of the accompanying vessel. A diagnosis of bronchiectasis is made on the basis of clinical symptoms (especially productive cough, often with a purulent component) consistent with radiological findings.^1^ ^,^ ^2^ There is usually a vicious circle of inflammation and chronic infection by pathogenic microorganisms, explaining symptom progression, radiological findings, and disease prognosis.^3^ Bronchiectasis has more than one hundred different causes, both pulmonary and extrapulmonary. In the 20th century, bronchiectasis was an “orphan” disease and was therefore not usually included in the differential diagnosis of chronic inflammatory diseases of the airway. However, since the beginning of the 21st century, large research groups worldwide have been collecting data on patients with bronchiectasis through national and international registries. There are currently 16 countries collecting complete individual datasets. In terms of number of patients, three registries stand out: the first, a national registry in China, with more than 16,000 patients since 2020; the second, a federal registry in the United States, with more than 8,000 patients since 2007; and the third, an international registry, the European Multicenter Bronchiectasis Audit and Research Collaboration registry, which includes 27 countries and more than 19,000 patients.^4^

PTLD has recently attracted considerable interest, given that approximately 50% of the human suffering attributed to tuberculosis occurs after successful completion of tuberculosis treatment.^5^ ^-^ ^7^ Patients continue to suffer from tuberculosis sequelae leading to a range of respiratory symptoms (including bronchiectasis) and nonrespiratory symptoms,^8^ ^-^ ^10^ accompanied by lung function decline,^11^ reduced quality of life, reduced exercise tolerance, and other complications, including a mortality rate that is five times higher than that for the general population.^8^ ^,^ ^10^ ^,^ ^11^

Little is known about how tuberculosis causes PTLD and bronchiectasis or how other infections can cause these two chronic respiratory conditions. The aforementioned registries allow us to gain a better understanding of the most common etiologies of bronchiectasis. The objective of the present study was to describe the prevalence of post-tuberculosis and postinfective bronchiectasis in different countries in the world and discuss the characteristics of both. 

Data from the existing bronchiectasis registries were obtained by personally contacting the coordinator of each registry or by reviewing the most updated published information. Data on post-tuberculosis bronchiectasis in different countries and registries were stratified into three groups on the basis of their proportions: low prevalence (< 10%), intermediate prevalence (10-15%), and high prevalence (> 15%). Proportions were compared by means of the chi-square test. A value of p ≤ 0.05 was considered significant. 

The combined data from all available registries are summarized in Table 1. Of 58,474 patients, 11.8% (range, 1.8-35.5%) had post-tuberculosis bronchiectasis and 27.3% (range, 19-43.2%) had postinfective bronchiectasis. 

**Table 1:** Total numbers and proportions of post-tuberculosis bronchiectasis and postinfective bronchiectasis in bronchiectasis registries worldwide. In orange, blue, and yellow, countries with a low, intermediate, and high prevalence of post-tuberculosis bronchiectasis, respectively.

Country or continent	Total number of patients	Period covered	Post-tuberculosis bronchiectasis, n (%)	Postinfective bronchiectasis, n (%)	Other causes of bronchiectasis, n (%)
Europe (EMBARC registry)	19,486	2015-present	955 (4.9%)	4,209 (21.6%)	73.5%
Australia	1,201	2016-present	22 (1.8%)	337 (28.1%)	70.1%
USA (BRR)	8,044	2014-present	322 (4%)	--	96%
Canada	289	2024-present	13 (4.5%)	33 (11.4%)	84.1%
Germany	1,989	1989-present	40 (2%)	378 (19%)	79%
Japan	1,597	2018-present	99 (6.2%)	431 (27%)	66.8%
Spain (RIBRON)	2,631	2015-present	355 (13.5%)	106 (40.4%)	32.6%
Turkey	1,035	2019-present	117 (11.3%)	409 (39.5%)	49.2%
China	16,389	2020-present	200 (12.2%)	708 (43.2%)	44.6%
Spain (SHBR)	2,123	2002-2011	390 (18.4%)	64 (30%)	51.6%
Argentina	617	2024-present	120 (19.4%)	160 (25.9%)	54.7%
South Korea	938	2015-present	189 (20.1%)	179 (19.1%)	60.8%
India	2,135	2015-2017	758 (35.5%)	478 (22.4%)	42.1%
TOTAL	58,474	range, 2002-2025	3,560 (11.8%)	7,492 (27.3%)	60.9%

EMBARC: European Multicenter Bronchiectasis Audit and Research Collaboration; BRR: Bronchiectasis and NTM (Nontuberculous Mycobacteria) Research Registry; RIBRON: *Registro Español Informatizado de Bronquiectasias* (Spanish Online Bronchiectasis Registry); and SHBR: Spanish Historical Bronchiectasis Registry.

Our data confirm that although idiopathic forms are the most prevalent,^5^ two forms of bronchiectasis are extremely common in almost all countries: post-tuberculosis bronchiectasis and postinfective bronchiectasis.^6^ ^,^ ^7^ Furthermore, although postinfective bronchiectasis remains fairly stable in terms of proportion (approximately 20-40%) and is undoubtedly the most common form of bronchiectasis, post-tuberculosis bronchiectasis shows greater heterogeneity, ranging from 1.8% in Australia to 35.5% in India. This may be due to how tuberculosis-related factors have acted over time, such as the extent of decline in tuberculosis incidence and the impact of tuberculosis control interventions, including the impact of tuberculosis infection management. The relationship between the decline in tuberculosis incidence and the proportions of post-tuberculosis bronchiectasis merits further investigation. 

In the case of postinfective bronchiectasis, it is more difficult to determine the etiology. Although the etiology of tuberculosis is bacteriologically confirmed, there is no objective evidence indicating that the cause of postinfective bronchiectasis is an infection, given that it is often attributed to childhood infections occurring several decades before. This means that if a patient had measles or whooping cough during childhood and developed bronchiectasis in adulthood, the bronchiectasis is frequently attributed to the childhood infection without an etiological diagnosis to rule out other potential causes. Perhaps the most reliable diagnosis is post-pneumonia bronchiectasis, given that there is objective imaging evidence of the pneumonic process, which is followed by the appearance of bronchiectasis in the same location. The fact that the aforementioned registries currently attribute bronchiectasis to a bacteriologically confirmed or clinically diagnosed case of infection is a limitation that requires attention to improve the quality of diagnosis of postinfective bronchiectasis. 

An interesting issue for reflection is that many studies examining the etiology of bronchiectasis include post-tuberculosis bronchiectasis in the group of postinfective bronchiectasis. This is probably not a good idea because although tuberculosis is a form of (mycobacterial) infection, it has important differentiating characteristics. 

There are many future challenges regarding the association of post-tuberculosis bronchiectasis and postinfective bronchiectasis with comorbidities,^12^ clinical presentation, and new therapeutic possibilities,^13^ ^,^ ^14^ given that the causative agents are of very different nature and could have different responses to treatment and different prognoses.^15^ Among the topics deserving further investigation, the role of rehabilitation in improving lung function, exercise capacity, and quality of life, as underscored in a recent study in Brazil,^16^ deserves special attention. 
